# Modeling and Optimization of Properties of the Environmentally Clean Molds Based on Oligofurfuryloxysiloxanes for the Production the Metal Castings

**DOI:** 10.3390/polym14091883

**Published:** 2022-05-05

**Authors:** Olga Ponomarenko, Nataliia Yevtushenko, Kristina Berladir, Mykola Zapolovskyi, Jan Krmela, Vladimíra Krmelová, Artem Artyukhov

**Affiliations:** 1 Department of Foundry, National Technical University “Kharkov Polytechnic Institute”, 2, Kyrpychova St., 61002 Kharkiv, Ukraine; 21ponomarenko@gmail.com; 2 Department of Occupational and Environmental Safety, National Technical University “Kharkov Polytechnic Institute”, 2, Kyrpychova St., 61002 Kharkiv, Ukraine; natalya0899@ukr.net; 3 Department of Applied Materials Science and Technology of Constructional Materials, Sumy State University, 2, Rymskogo-Korsakova St., 40007 Sumy, Ukraine; 4 Department of Computer Engineering and Programming, National Technical University “Kharkov Polytechnic Institute”, 2, Kyrpychova St., 61002 Kharkiv, Ukraine; zapolovsky@email.ua; 5 Faculty of Mechanical Engineering, J. E. Purkyně University in Ustí nad Labem, Pasteurova 1, 400 96 Ustí nad Labem, Czech Republic; jan.krmela@ujep.cz; 6 Faculty of Industrial Technologies in Púchov, Alexander Dubček University of Trenčín, Ivana Krasku 491/30, 02001 Púchov, Slovakia; vladimira.krmelova@tnuni.sk; 7 Academic and Research Institute of Business, Economics and Management, Sumy State University, 2, Rymskogo-Korsakova St., 40007 Sumy, Ukraine; a.artyukhov@pohnp.sumdu.edu.ua

**Keywords:** cold hardening polymer composition, oligofurfuryloxysiloxanes, catalyst, strength, survivability, computational modeling, mathematical dependencies, energy efficiency

## Abstract

This article is devoted to modeling, researching and optimizing the main properties of an environmentally clean polymer composition based on oligofurfuryloxysiloxanes (OFOS), which can be used to produce casting molds and cores in the production of castings from ferrous and nonferrous metals. Polymer compositions were examined for strength, survivability, gas permeability, moisture, crumbliness, fire resistance, knockout, and stickability. It has been established that the increase in the strength of the polymer composition over time obeys an exponential law. Mathematical equations were derived for all the exponential curves. The indications of compressive strength of the polymer composition with OFOS with all the acid catalysts used were, on average, as follows: after 1 h—1.3–1.54 MPa; after 3 h—2.5–2.9 MPa; after 24 h—4.9–6.1 MPa, which meets the requirements for casting molds before pouring with metal. The use of polymer compositions with OFOS ensures environmental safety of the technological process, due to the lack of emission of toxic substances, both in the “cold” stage of the process and during casting with molten metal, cooling, knocking out, and disposal of polymer compositions. This makes it possible to save energy resources, and thereby reduce the total cost of the entire technological process and castings.

## 1. Introduction

Cast metal products account for a significant share in labor intensity and weight for any mechanical engineering product. The competitiveness of the final products (engines, machine tools, automobiles, etc.) is determined by the quality of castings, their accuracy and production efficiency. The improvement of mechanical engineering products occurs by increasing the complexity, accuracy, quality, performance, reducing the thickness of the walls and the mass of cast parts, additive casting applications etc. [1,2]. In recent years, the metal consumption of products has been reduced by 10–20% [3], and casting allowances and tolerances have been reduced by more than 1.5–2.0 times [4].

The increasing complexity, accuracy and reduction in thin-walled and cast parts, along with the requirements of minimizing labor costs and effective environmental protection, significantly affect the development of technologies for the production of castings. Senthil et al. [5] proposed vacuum casting, which allows manufacturers to eliminate sand mixers and other equipment required for hardened sand. This process is generally clean and free of fumes or odors and the dry sand is simply recycled for the next application. Kuo et al. [6] explored a type of technology to develop an injection mold with high cooling performance, by integrating molding simulation and rapid tooling technology. It was found that using polyurethane foam for a large injection mold saved about 50% of the production cost. Gacs et al. [7] investigated an adhesion property of the different polymer surfaces that are treated with various plasma processes parameters. They used a 2^n−1^ factorial design of experiments study and observed no correlation between either part of the surface free energy and the adhesion properties of the prepared surfaces. Rajaguru et al. [8] developed an indirect rapid tooling using electroless nickel plating for low-volume production of plastic injection moldings for the rapid prototyping models. The cavity insert can be fabricated using commonly available low-cost materials within 48 h and the tool life is longer under normal plastic processing conditions. Liu et al. [9] proposed a new water-soluble calcia-based ceramic core, using epoxy resin binder for investment casting by aqueous gel. It is reported that such a material could overcome the poor leachability of the common ceramic core and enhanced the production efficiency. Kuo et al. [10] also experimentally investigated the effects of four different coolant media on the cooling performance of ten sets of injection molds fabricated with different mixtures. It was found that the epoxy resin with 41 vol.% aluminum powder was the optimal formula for making an injection mold, since a saving in the total production cost of about 24% is made, compared to the injection mold made with commercially available materials.

The essence of obtaining castings is that the molten and superheated alloy of a given composition is poured into a pre-prepared mold, the inner cavity of which with the maximum degree of approximation reproduces the configuration and dimensions of the future product [11]. The melt, filling the inner cavity of the mold, crystallizes in it and forms a casting. The outer contours of the casting are formed by the walls of the mold cavity, and the internal cavities, holes, cavities, channels and complex outer surfaces are formed by inserts into the molds called rods.

To obtain a casting free from defects, molds must meet a set of certain properties. Gutowski and Błędzki [12] presented the optimized composite materials with increased thermal resistance temperature, which allow for the flexible control of material properties and fabrication. Hamasaiid et al. [13] investigated two aluminum alloys and established that the apparent solidification time of Al-9Si-3Cu is larger than that of Al-7Si-0.3Mg and it increases with coating thickness.

The main volume of the obtained castings (75–80%) is made in one-time forms, the properties of which determine the quality of the castings. The occurrence of surface casting defects [14] is due to the unsatisfactory quality of the molding materials and mixtures from which the mold is made. Chen and Kaufmann [15] proposed an approach for the application of machine learning in the prediction and understanding of casting surface related defects. They developed six different machine learning algorithms by which production data from a steel and cast-iron foundry can be used to create models for predicting casting surface-related defects.

The processes of making molds, which occupy an important place in the production of castings, are constantly being improved. Grimzin et al. [16] developed a technology for the preparation and drying of molds and cores based on sand–plaster mixtures. It determined the values of strength, durability, gas permeability, friability that make up the quality of a casting. The strength of the mold must be such that the configuration and dimensions of the mold do not change during the process of filling with the melt, during crystallization [17] and for some time during its subsequent cooling [18], until the poured metal acquires sufficient strength to maintain the configuration of the casting. However, the strength should not be high during the shrinkage of the casting, or the mold’s resistance to shrinkage will cause stresses and cracks in the castings. These two mutually opposing requirements can be met in the manufacture of molds based on polymer compositions.

Today, a greater number of casting molds are made using cold-hardening mixtures (CHM) based on polymer compositions in single and serial production in industrialized countries. Ponomarenko et al. [19] proposed a new method of manufacturing cores based on CHM, which harden at room temperature. This is due to the high strength of the mixture at low consumption, the ability to control the curing rate of the mixture in a wide range [20], the absence of a drying operation and the need for drying equipment [21], easy knocking out of mixtures from the internal cavities of castings and castings from molds [22], low energy consumption of the process, which greatly simplifies and reduces the casting manufacture cycle [23].

However, one significant problem is the negative impact of the products of thermal degradation of polymer compositions on the safety of human life and the environment [24]. When used, from 30 to 40% (by mass) of toxic products are formed in the form of gases and condensate [25]. A significant part of the degradation products remains in the waste mixtures. Today, it is almost impossible to refuse CHM based on polymer compositions in the foundry industry. Therefore, the development and application of environmentally clean compositions of cold-hardening mixtures for casting molds, based on polymer compositions with preservation of the indicators of their basic physical, mechanical and technological properties, and the development of their preparation technology is the actual problem with the production of cast parts.

The paper aims to model, research and study the main properties of an environmentally clean polymer composition based on oligofurfuryloxysiloxanes (OFOS), which can be used to produce casting molds and cores in the production of castings from ferrous and nonferrous metals.

Acid-curing synthetic polymers (resins) are products of the polycondensation of formaldehydes with carbamide (urea) and/or phenol and/or furfuryl alcohol and include the following: urea-formaldehyde (urea) resins; urea-formaldehyde-furan (urea-furan) resins; urea-phenol-formaldehyde (urea-phenolic) resins; phenolcarbamide-formaldehyde (phenolcarbamide) resins; phenol-formaldehyde (phenolic) resins and phenol-formaldehyde-furan (phenol-furan) resins. The polymer composition based on OFOS also cures under the action of acids. However, the use of polymer compositions with OFOS ensures the environmental safety of the process, due to the absence of release of toxic substances, both in the “cold” stage of the process and during pouring with molten metal, cooling, knocking out and disposal of polymer compositions.

The main research objectives are to develop and optimize the mathematical models of technological process for obtaining environmentally clean casting molds based on polymer compositions.

## 2. Materials and Methods

Polymer compositions, consisting of an oligomer, hardener or catalyst and filler, are used to make the molds. The curing is carried out by a polycondensation reaction, which continues in the CHM with the addition of a catalyst.

We use an environmentally clean binder based on oligofurfuryloxysiloxanes (OFOS) as an oligomer in the production of the casting molds. OFOS is a mobile liquid of a dark brown color, which solidifies under the action of acid hardeners. In its composition, OFOS does not contain diphenylolpropane, toxic or poisonous substances (such as phenols and aldehydes), which are released during thermal destruction during the pouring of molds with molten metal.

During hardening, a polymer composition is formed with a spatial network structure where, along with longitudinal bonds, their macromolecules also have cross-links. This structure provides high mold strength.

OFOS can be obtained in various modifications, containing 4 to 6 moles of furfuryloxy groups. In this work, MF4, MF5, MF6 oligomers containing 4, 5, 6 furfuryloxy groups, respectively, are studied. Ethyl silicate-40 (ETS-40), furfuryl alcohol and a catalyst are used to obtain OFOS.

**Figure 1:**
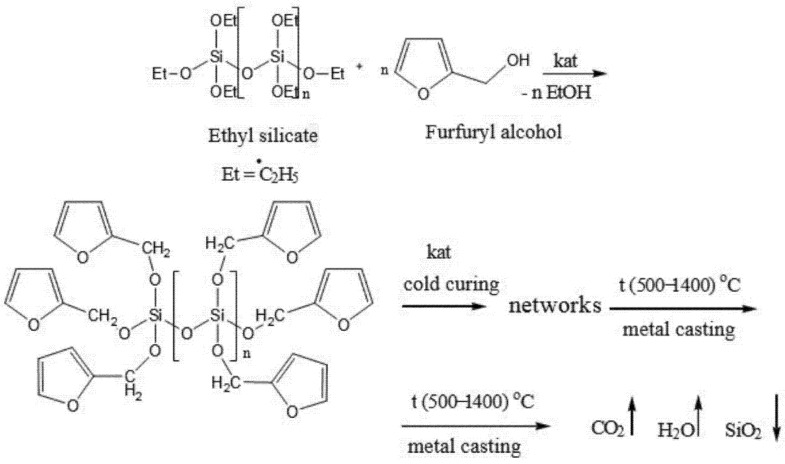
Structural scheme of processes that occur in polymer composition.

The processes that occur in the polymer composition are structurally described in Figure 1.

An ion-radical mechanism polymerizes the polymer composition, by revealing double bonds in furan cycles at normal room temperatures. At the same time, it heats up to a temperature of 60...70 °C, due to the heat of polymerization of double bonds and forms a mesh structure in conditions of cold molding of molds and cores. The interaction of the components with the oligomer OFOS does not form free furfuryl alcohol, as, for example, when using furan resins.

The thermal destruction of the mesh polymer composite binder occurs when pouring the molten metals into the molds. As a result of thermal destruction, CO_2_ and H_2_O vapors are released into the atmosphere, and an inorganic solid residue SiO_2_ is formed, which can be reused.

The technology of manufacturing the molds and rods using these polymer compositions ensures the environmental safety of the technological process, due to the absence of toxic and toxic substances, both in the “cold” stage of the process when pouring the molten metal, cooling, embossing, and disposal of molding mixtures. It eliminates the operation of knocking the cores out of the castings and castings out of the molds, making it possible to use low-cost tooling. In addition, their use makes it possible to save energy resources, and thereby reduce the total cost of the entire technological process and the target product for the castings.

The quality of the casting mold is determined by the quality of the molding polymer compositions used in their manufacture.

In this work, the main properties of polymer compositions were studied, on which the quality of castings during sand casting depends. These include strength, survivability, gas permeability, moisture, crumbliness, refractoriness, knockout, and stickability. The methods for studying the physical-mechanical and technological properties of the polymer composition were based on standard research methods.

The polymer composition for making the molds was prepared in the following way. Per 100 w.p. of quartz sand 2K1O302, 1–2 w.p. of 50–70% aqueous acid solution was added. Benzenesulfonic acid (BSA), p-toluenesulfonic acid (PTSA) and sulfosalicylic acid (SSA) were used in this work. The components were thoroughly mixed for 60 s, then 1.2–2 w.p. of OFOS was added and again thoroughly mixed for 120 s. Different modifications of the OFOS with 4 to 6 moles of furfuryloxy groups were used.

The resulting polymer composition was molded into a 9-seat mold, kept for 30 min, and the samples were removed from the mold.

## 3. Results and Discussions

### 3.1. Modeling of Compressive Strength of Polymer Compositions

Based on the experiments, it was found that the strength readings, according to the technological test for compression of the polymer composition with OFOS with all the acid catalysts used, were, on average, as follows: after 1 h—1.3–1.54 MPa; after 3 h—2.5–2.9 MPa; after 24 h—4.9–6.1 MPa, which meets the requirements for casting molds before pouring with metal.

Of interest is the information on the increase in compressive strength of the polymer composition in the initial time period. It is primarily due to the opening time of the core box or the time when the mold is ready for pouring. This is one of the most significant technological and economic parameters in the foundry to produce molds and cores. During this time, the “lag” in forming the strength of the polymer composition of the inner cores of the rod or molds is eliminated.

**Figure 2:**
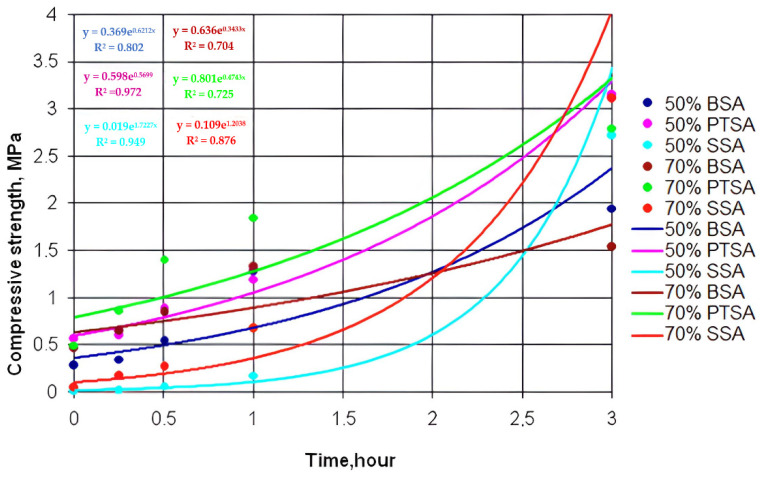
Kinetic dependences of the increase in the strength of the polymer composition using MF4 type OFOS in the presence of various catalysts.

**Figure 3:**
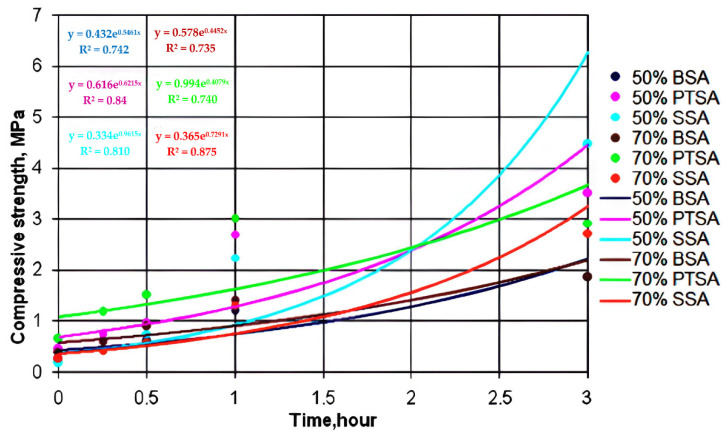
Kinetic dependences of the increase in the strength of the polymer composition using MF5 type OFOS in the presence of various catalysts.

**Figure 4:**
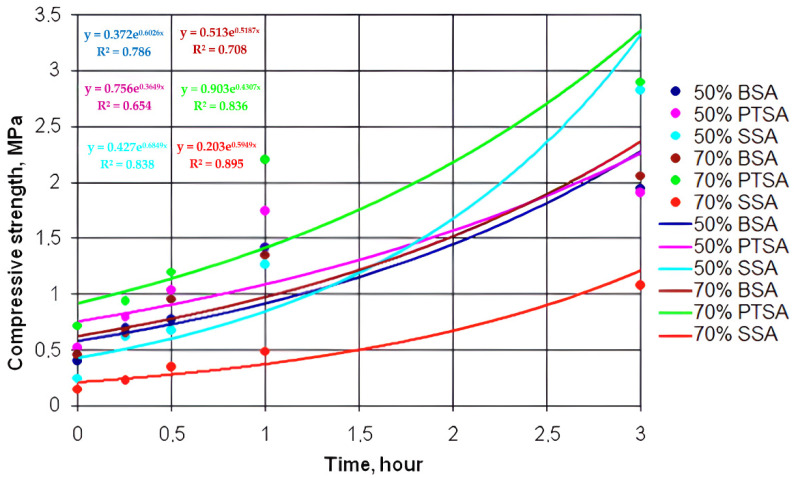
Kinetic dependences of the increase in the strength of the polymer composition using MF6 type OFOS in the presence of various catalysts.

Figure 2, Figure 3 and Figure 4 show the dependence of the increase in the compressive strength of the polymer composition for three hours using MF4, MF5, andMF6 types of OFOS in various acid catalysts.

Graphic dependences were obtained by exponential approximation of the data using the least squares method according to the following equation:

Y = C×ebx,(1)

where c, b—constants,

e—base of natural logarithm.

As a result of data processing in the studied interval, it was found that the increase in the strength of the polymer composition over time obeys an exponential law. For all the exponential curves, mathematical dependences were obtained.

Table 1 shows the mathematical dependences of the increase in the strength of the polymer composition with OFOS and the magnitude of the approximation reliability R^2^.

**Figure 5:**
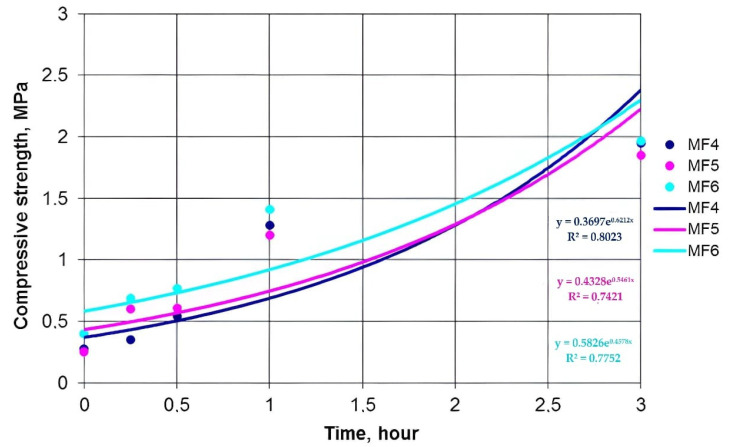
Comparative characteristics of the polymer composition OFOS type MF4, MF5, MF6 with a catalyst 50% BSA.

Figure 5 shows the exponential dependences of the increase in the strength of the polymer composition using MF4, MF5, MF6 type OFOS in the presence of a 50% BSA catalyst.

The establishment of the patterns of the increase in the strength of the polymer composition and their graphical representation makes it possible to present information in a simple and visual form. For example, from comparing the strength of a polymer composition with different oligomers (MF4, MF5, and MF6), we can conclude that an increase in the degree of polymerization n leads to an increase in its strength, regardless of the catalyst.

A dependence analysis showed that the strength of the samples of the polymer composition increases with decreasing catalyst concentration, i.e., the strength of the samples with a catalyst and with an acid content of 50% is higher than with a catalyst content of 70%. This is due to the high activity of hydrogen ions because, in the composition of 50% of the catalyst, there is more water than in the composition of 70% of the catalyst. At normal temperatures (t = 25–30 °C), sand grains have a more uniform envelopment, and the reaction with OFOS is faster.

The analysis of the data indicates that the samples with the 50% PTSA catalyst in this time period have a higher compressive strength than the samples with the 50% BSA catalyst. The initial compressive strength of the specimens with the SSA catalyst increases more slowly than that of the specimens with the BSA and PTSA catalysts.

The main method for regulating the survivability of a polymer composition is to change the degree of polymerization n of the resin and its quantity, concentration, and consumption of the catalyst solution. Ceteris paribus, the survivability of the polymer composition is the lower that the higher the temperature of the materials and the environment.

The curing speed determines the pot’s life. It depends mainly on the polymer composition, the quality of the raw materials and their temperature, the ambient temperature, and the intensity and duration of mixing. Data analysis showed that the survivability of mixtures based on MF4, MF5, and MF6 in the presence of BSA, PTSA, and SSA catalysts is within 4–17 min. Moreover, an increase in the concentration of the catalyst leads to a decrease in survivability. The gas-forming capacity averages 10.5–11.8 cm^3^/g, which is a good indicator for polymer compositions in manufacturing molds. The gas permeability of the polymer composition is >200 units. The shedding of all-polymer compositions is insignificant and is in the range of 0.1–0.36%.

The moisture content of the polymer composition depends on the concentration of the catalyst in the following way. With an increase in the concentration of the catalyst, its moisture content decreases. The moisture content of the polymer composition ranges from 0.02 to 0.06%. Knockout is good because during thermal degradation, the OFOS is completely burned out. The polymer composition in the mold near the casting and gating systems crumbles, and the cores are usually completely or partially destroyed. The adhesion of the polymer composition to the core box and model is minimal, and the burn-in is negligible.

### 3.2. Modeling of Optimal Parameters and Properties of Polymer Composition

A planned experiment was carried out to simulate the properties of a polymer composition based on OFOS.

For the optimization parameters (y), the polymer composition properties’ leading physical and mechanical indicators were chosen—compressive strength and survivability (y_1_ and y_2_, respectively). The polymer compositions with different percentages of oligomers and catalysts were studied.

The following variable factors were chosen: the amount of OFOS oligomer introduced (x_3_), the amount (x_1_), and the concentration (x_2_) of the catalyst used. PTSA was used as a catalyst.

The intervals of the variation in the factors and their values at the main, upper and lower levels are shown in Table 2.

The planning matrix for experiments 2^6−3^ is shown in Table 3. The mathematical model considered the influence of variable factors and their paired interactions.

To exclude the influence of systematic errors caused by external conditions, the studies specified by the planning matrix were carried out in a random order, randomized in time. The order of the studies was chosen according to the table of random numbers.

The experimental error was considered during the experiment, i.e., reproducibility variance. Reproducibility variance was assessed from the results of parallel studies. Each investigation in the planning matrix was carried out three times to do this.

As a result of processing the experiments, the following system of equations was obtained:

y_1_ = 0.85 + 0.44x_1_ − 0.2x_2_ + 0.07x_3_ + 0.12x_1_x_3_ [MPa], (2)

y_2_ = 6.6 − 1.4x_1_ − 1.3x_2_ + 1.3x_3_ [min], (3)

The obtained equations can be used to evaluate the influence of the input parameters on the properties of the polymer composition and optimize it.

Based on the mathematical models on a technological basis (curing cycle), the following two groups of compositions have been developed: a polymer composition with a normal curing cycle OFOS-N (20–40 min) and an accelerated curing cycle OFOS-S (5–10 min).

The resulting equations can be used to optimize the composition of the polymer composition. The resulting system of Equations (1) and (2) was taken as a basis.

When analyzing the obtained data, it was established that the influence of the variable factors on the optimization parameters corresponds to the theoretical ideas about the formation of the properties of the polymer composition during its preparation.

The strength of the polymer composition increases with an increase in the amount of OFOS and the amount of PTSA catalyst. It has been established that, at the studied concentrations and normal temperature, the lower the catalyst concentration, the higher the strength of the polymer composition.

The survivability of the polymer composition decreases with an increase in the amount of catalyst and a decrease in the amount of OFOS. The process parameters of the polymer composition are also affected by the pairwise interactions of the initial components.

Based on the developed mathematical models, a nomogram was constructed that describes the relationship between the technology parameters and the polymer composition properties. The nomogram is a means of a graphical solution to this problem.

**Figure 6:**
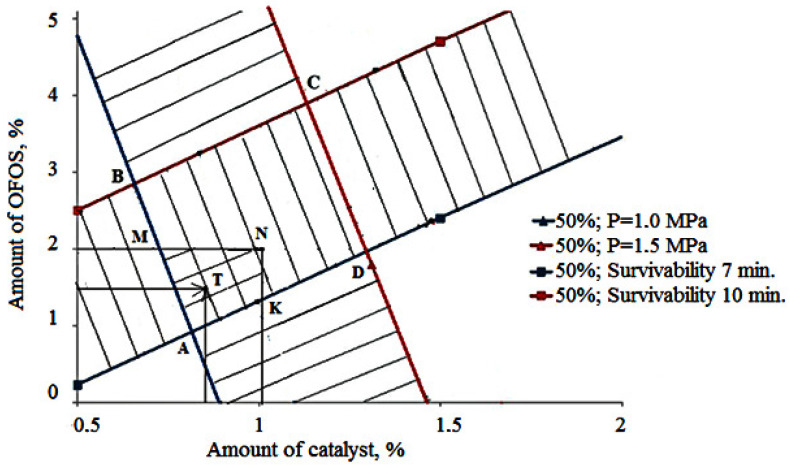
Nomogram for determining the optimal composition of the polymer composition based on OFOS at a catalyst concentration of 50%: AB—direct line for which the compressive strength is 1.0 MPa; CD—direct line for which the compressive strength is 1.5 MPa; AD—direct line for which survivability is 7 min; BC—direct line for which survivability is 10 min; ABCD—area that meets the requirements for the quality of the polymer composition; AMNK—the area of optimal values for the properties of the polymer composition.

When constructing the nomogram, it was considered that, for production conditions, the compressive strength should be in the range from 1.0 MPa to 1.5 MPa, and the survivability should be from 7 to 10 min. The nomogram is shown in Figure 6. Line AB represents the set of points for which the compressive strength is 1.0 MPa, and line CD is 1.5 MPa. Similarly, direct AD is 7 min for survivability, and direct BC is 10 min. Quadrilateral ABCD is a set of points that meet the requirements for the quality of the polymer composition. If we assume that using the OFOS oligomer of more than 2.0% and the catalyst of more than 1.0% is considered an irrational use of materials the region of optimal values for the properties of the composite is the region described by the AMNK polygon.

For example, point T on the nomogram shows that, when used in a mixture of OFOS in an amount of 1.5% and a catalyst of 0.8%, the mixture will meet the production requirements. Point A shows the minimum number of components that must be taken to obtain a polymer composition of the required quality.

Based on the data of the nomogram, it is possible to adjust the parameters of the polymer composition preparation process by changing the number of components, which can be used to quickly control the properties of the polymer composition.

The use of a nomogram makes it possible to stabilize the properties of a cold-hardening polymer composition under industrial conditions and makes it possible to carry out the following:

- To predict the strength and survivability of a cold hardening polymer composition;
- For a given strength or survivability, determine the required compound of a cold hardening polymer composition.

A technological process has been developed to prepare core and molding cold-hardening polymer compositions based on OFOS to obtain high-quality castings. The cold hardening polymer composition, which satisfies the requirements for the quality of castings, is determined by the following range of values: for the OFOS oligomer, it is from 1.0% to 2.0% and for the PTSA catalyst, it is from 0.6% to 1.0%.

The prospect of using the proposed polymers lies in the fact that it is not required to change the technological process of manufacturing molds for obtaining the castings that exist at the enterprise for their use. However, the production of these polymers has not yet been put on an industrial basis.

Further studies suggest modifying the compositions of OFOS polymers to be able to use them to produce massive castings from high-alloy steels, with a melting point above 1500 °C.

## 4. Conclusions

The polymer compositions based on OFOS were investigated for survivability, gas-holding capacity, moisture, gas permeability, crumbling, flame retardancy, and knockout. The analysis of the obtained results of the work showed that the content of OFOS in the polymer composition is the leading indicator of design. It determines the strength characteristics of cores and molds, quality of castings, sanitary and hygienic characteristics of the process, and its technical and economic efficiency. An increase in polymerization measure leads to an increase in strength.

The main method of controlling survivability is to change the degree of resin polymerization and the catalyst solution’s amount, concentration, and consumption. Increasing the concentration of the catalyst and the degree of polymerization of the resin leads to a decrease in the survivability of the polymer composition. Other things being equal, the lower the survivability of the mixture, the higher the temperature of the materials and the environment.

It was found that the strength of the polymer composition increases with decreasing catalyst concentration, i.e., its strength with a catalyst containing 50% acid is higher than with 70% acid, which is due to the high activity of hydrogen ions. The analysis of the data shows that the polymer composition with the PTSA catalyst has a higher compressive strength than the samples with the BSA and SSA catalyst.

A planned experiment was carried out to simulate the properties of a polymer composition based on OFOS. The following can be noted: the influence of variable factors on the optimization parameters corresponds to the theoretical ideas about forming the properties of a cold-hardening polymer composition during its preparation.

The strength of the polymer composition increases with an increase in the amount of OFOS and the amount of PTSA catalyst. It has been established that, at the studied concentrations and normal temperature, the lower the catalyst concentration, the higher the strength of the polymer composition.

A technological process has been developed to prepare core and molding cold-hardening polymer compositions based on OFOS to obtain high-quality castings. The cold hardening polymer composition, which satisfies the requirements for the quality of castings, is determined by the following range of values: for the OPOS oligomer, it is from 1.0% to 2.0% and for the PTSA catalyst, it is from 0.6% to 1.0%.

Based on the mathematical models on a technological basis (curing cycle), the following two groups of compositions have been developed: mixtures with a normal curing cycle OFOS-N (20−40 min) and an accelerated curing cycle OFOS-S (5−10 min).

Further studies suggest modifying the compositions of OFOS polymers to be able to use them to produce massive castings from high-alloy steels, with a melting point above 1500 °C.

## Figures and Tables

**Table 1:** Mathematical dependences of the increase in the strength of the polymer composition with OFOS on the type and concentration of the catalyst.

Acid Catalyst	Formula for OFOS Type		
	MF4	MF5	MF6
50% BSA	y = 0.3697 e^0.6212x^ R^2^ = 0.8023	y = 0.4328 e^0.5461x^ R^2^ = 0.7421	y = 0.3724 e^0.6026x^ R^2^ = 0.7861
50% PTSA	y = 0.5985 e^0.5699^ R^2^ =0.9728	y = 0.6168 e^0.6215x^ R^2^ = 0.84	y = 0.756 e^0.3649x^ R^2^ = 0.6543
50% SSA	y = 0.0196 e^1.7227x^ R^2^ = 0.9491	y = 0.3349 e^0.9615x^ R^2^ = 0.8102	y = 0.4277 e^0.6849x^ R^2^ = 0.8381
70% BSA	y = 0.6361 e^0.3433x^ R^2^ = 0.7048	y = 0.578 e^0.4452x^ R^2^ = 0.7354	y = 0.5137 e^0.5187x^ R^2^ = 0.7081
70% PTSA	y = 0.8012 e^0.4743x^ R^2^ = 0.7257	y = 0.9947 e^0.4079x^ R^2^ = 0.7404	y = 0.9036 e^0.4307x^ R^2^ = 0.8362
70% SSA	y = 0.1098 e^1.2038^ R^2^ = 0.8767	y = 0.3659 e^0.7291x^ R^2^ = 0.8751	y = 0.2034 e^0.5949x^ R^2^ = 0.895

**Table 2:** Conditions for conducting experiments to obtain a polymer composition based on OFOS.

Factors	Amount of Acid, %	Concentration of Acid, %	Amount of OFOS, %
The code	x_1_	x_2_	x_3_
Main level	1	60	2
Variation interval	0.5	10	1
Top level	1.5	70	3
Lower level	0.5	50	1

**Table 3:** Planning matrix and indicators of initial parameters.

Number of Experiences	Quantity of PTSA (x_1_)	Concentration of PTSA (x_2_)	Quantity of OFOS (x_3_)	x_0_	x_1_	x_2_	x_3_	x_1_x_2_	x_1_x_3_	x_2_x_3_	y_1_	y_2_
1	1.5	70	3	+	+	+	+	+	+	+	1.22	5
2	0.5	70	3	+	–	+	+	–	–	+	0.23	9
3	1.5	50	3	+	+	–	+	–	+	–	1.75	7
4	0.5	50	3	+	–	–	+	+	–	–	0.49	10
5	1.5	70	1	+	+	+	–	+	–	–	0.92	3
6	0.5	70	1	+	–	+	–	–	+	–	0.20	5
7	1.5	50	1	+	+	–	–	–	–	+	1.29	6
8	0.5	50	1	+	–	–	–	+	+	+	0.72	8

## Data Availability

The data presented in this study are available on request from the corresponding author.

## Nomenclature

CHM	Cold-hardening mixtures
OFOS	Oligofurfuryloxysiloxanes
ETS-40	Ethyl silicate-40
BSA	Benzenesulfonic acid
PTSA	p-toluenesulfonic acid
SSA	Sulfosalicylic acid

## References

[1] Sivarupan T., Balasubramani N., Saxena P., Nagarajan D., Mansori M.E., Salonitis K., Jolly M., Dargusch M.S. (2021). A review on the progress and challenges of binder jet 3D printing of sand moulds for advanced casting. Addit. Manuf..

[2] Prakash C., Singh S., Kopperi H., Ramakrihna S., Mohan S.V. (2021). Comparative job production based life cycle assessment of conventional and additive manufacturing assisted investment casting of aluminium: A case study. J. Clean. Prod..

[3] Lubimyi N.S., Polshin A.A., Gerasimov M.D., Tikhonov A.A., Antsiferov S.I., Chetverikov B.S., Ryazantsev V.G., Brazhnik J., Ridvanov İ. (2022). Justification of the Use of Composite Metal-Metal-Polymer Parts for Functional Structures. Polymers.

[4] Nayak R.K., Venugopal S. (2018). Prediction of shrinkage allowance for tool design of aluminium alloy (A356) investment casting. Mater. Today Proc..

[5] Senthil J., Prabhahar M., Thiagarajan C., Prakash S., Lakshmanan R. (2020). Studies on performance and process improvement of implementing novel vacuum process for new age castings. Mater. Today Proc..

[6] Kuo C.-C., Nguyen T.-D., Zhu Y.-J., Lin S.-X. (2021). Rapid Development of an Injection Mold with High Cooling Performance Using Molding Simulation and Rapid Tooling Technology. Micromachines.

[7] Gacs J., Vernon Z., Kocsis L., Berényi Z.J., Bogya E.S., Jacob T. (2022). Epoxy mold adhesion on various plasma-treated thermoplastic polymer surfaces. Int. J. Adv. Manuf. Technol..

[8] Rajaguru J., Duke M., Au C. (2015). Development of rapid tooling by rapid prototyping technology and electroless nickel plating for low-volume production of plastic parts. Int. J. Adv. Manuf. Technol..

[9] Liu F., Fan Z., Liu X., He J., Li F. (2016). Aqueous gel casting of water-soluble calcia-based ceramic core for investment casting using epoxy resin as a binder. Int. J. Adv. Manuf. Technol..

[10] Kuo C.-C., Xu J.-Y., Zhu Y.-J., Lee C.-H. (2022). Effects of Different Mold Materials and Coolant Media on the Cooling Performance of Epoxy-Based Injection Molds. Polymers.

[11] Mohanty U.K., Sarangi H., Abdallah Z., Aldoumani N. (2021). Solidification of Metals and Alloys. Casting Processes and Modelling of Metallic Materials.

[12] Gutowski W.S., Błędzki A.K. (2021). Fast-Setting Permeable Alkyd/Polyester Composites: Moulding Sands. Polymers.

[13] Hamasaiid A., Dargusch M.S., Davidson C., Tovar S., Loulou T., Rezai-Aria F., Dour G. (2007). Effect of Mold Coating Materials and Thickness on Heat Transfer in Permanent Mold Casting of Aluminium Alloys. Metall. Mater. Trans. A.

[14] Prabhakar A., Papanikolaou M., Salonitis K., Jolly M. (2020). Minimising Defect Formation in Sand Casting of Sheet Lead: A DoE Approach. Metals.

[15] Chen S., Kaufmann T. (2022). Development of Data-Driven Machine Learning Models for the Prediction of Casting Surface Defects. Metals.

[16] Grimzin I., Ponomarenko O., Marynenko D., Yevtushenko N., Berlizeva T. The Technological Process of Obtaining Sand-Plaster Molds for Complex Thin-Walled Aluminum Castings. Proceedings of the Grabchenko’s International Conference on Advanced Manufacturing Processes (InterPartner-2019).

[17] Aliotta L., Sciara L.M., Cinelli P., Canesi I., Lazzeri A. (2022). Improvement of the PLA Crystallinity and Heat Distortion Temperature Optimizing the Content of Nucleating Agents and the Injection Molding Cycle Time. Polymers.

[18] Mohapatra S., Sarangi H., Kumar U. (2020). Mohanty Effect of processing factors on the characteristics of centrifugal casting. Manuf. Rev..

[19] Ponomarenko O., Grimzin I., Yevtushenko N., Lysenko N., Marynenko D. Advanced Technologies of Manufacturing Readily Removable Cores for Obtaining High-Quality Castings. Proceedings of the 4th International Conference on Design, Simulation, Manufacturing: The Innovation Exchange, DSMIE-2021.

[20] Thu A.H., Zakharov A.I. (2018). Preparation of Inorganic Binder for Cold-Hardening Mixtures. Refract. Ind. Ceram..

[21] Akimov O., Penzev P., Marynenko D., Saltykov L. (2018). Identification of the behavior of properties of a cold-hardening glass-liquid mixture with propylene-carbonate different in dosing components. Technol. Audit Prod. Reserves.

[22] Körber S., Moser K., Diemert J. (2022). Development of High Temperature Resistant Stereocomplex PLA for Injection Moulding. Polymers.

[23] Czarnecka-Komorowska D., Grześkowiak K., Popielarski P., Barczewski M., Gawdzińska K., Popławski M. (2020). Polyethylene Wax Modified by Organoclay Bentonite Used in the Lost-Wax Casting Process: Processing−Structure−Property Relationships. Materials.

[24] Webb H.K., Arnott J., Crawford R.J., Ivanova E.P. (2013). Plastic Degradation and Its Environmental Implications with Special Reference to Poly(ethylene terephthalate). Polymers.

[25] Chamas A., Moon H., Zheng J., Qiu Y., Tabassum T., Jang J.H., Abu-Omar M., Scott S.L., Suh S. (2020). Degradation Rates of Plastics in the Environment. ACS Sustain. Chem. Eng..

